# Descriptive Study of Conjunctival Cysts: A Rare Complication after Strabismus Surgery

**DOI:** 10.1155/2018/1076818

**Published:** 2018-06-19

**Authors:** Xiaoshan Min, Hui Jiang, Lingyan Shi

**Affiliations:** ^1^Department of Ophthalmology, Xiangya Hospital, Central South University, Changsha, Hunan Province, China; ^2^Department of Ophthalmology, The Second Affiliated Hospital of Hunan University of Chinese Medicine, Changsha, Hunan Province, China

## Abstract

**Aim:**

Conjunctival cyst is one of the uncommon complications of strabismus surgery. It is important for surgeons and patients to be aware of and take precautions to minimize the risk. This study aimed to explore the clinical manifestations, etiology, and prognosis of conjunctival cyst at the operative site after strabismus surgery.

**Methods:**

The data of 1675 patients were included in our retrospective analysis, who underwent strabismus surgery at the Xiangya Hospital of Central South University between 2010 and 2016. During the postoperative follow-up, conjunctival cyst was found in 7 cases (7 eyes; 0.4% detective rate of all cases). The clinical characteristics, prognosis, and follow-up data were recorded together with the results of pathological and bacteriological tests.

**Results:**

Seven patients between the age of 3 years 8 months and 39 years, with the mean age of 12.71 years (12.71 ± 12.59, years of age), were included in the study. Strabismus surgery affected 13 recti, 8 medial and 5 lateral recti, and 3 obliques (all inferior oblique). Conjunctival cyst was detected in seven patients between 10 days and 6 months postoperatively (42.57 ± 61.11, detected days). In six cases, the cyst was detected at the nasal (3 cases) or temporal side (other 3 cases), and at the fornix in one case. Four out of 7 patients underwent cyst excision, and methicillin-resistant Staphylococcus aureus (MRSA) was detected in one patient.

**Conclusions:**

Conjunctival cyst is a rare postoperative complication of strabismus surgery, conjunctival epithelium implantation should be the primary cause, and infection might exaggerate the situation. A longer duration of the surgical procedure could increase the possibility of infection, which could be accompanied with a greater tendency to the occurrence of conjunctival cyst.

## 1. Introduction

Strabismus is a common ocular disorder occurring at all ages, with an estimated prevalence of 2–5% in the general population [1–4]. The purpose of strabismus treatment is to improve ocular alignment and make bilateral eye movement concordant, as well as to recover or rebuild comfortable binocular vision [5]. Surgery is a common and effective cure for treatment of strabismus [6]. Combined with glasses, prisms-wearing, and visual training before and after surgery, strabismus surgery creates the opportunity of building and restoring binocular vision function and eventually improves the possibility of increasing of visual acuity, or even patients' quality of life [7, 8]. Strabismus surgery is also called extraocular muscle surgery; it is a minimally invasive surgery under direct vision, with limited complications and quick recovery [9].

Conjunctival cyst is a rare complication after strabismus surgery; according to the time of onset, it is primarily caused by infection, conjunctival epithelial implantation, and chronic allergic response (probably a response to the suture). It typically manifests as a conjunctival abscess, granuloma or an epithelial inclusion cyst, and chronic or nonspecific inflammation [10, 11]. Most conjunctival cysts subside spontaneously, whereas surgery should be considered for cases not going into remission after a long period of time or those with symptoms, such as foreign body sensation, redness, swelling, hot, and painful eye [10, 11]. In this report, clinical data for 7 cases of conjunctival cyst, recorded by a strabismus surgeon at the Department of Pediatric Ophthalmology, Xiangya Hospital of Central South University, were analyzed. The purpose of the study was to utilize the data and summarize the existing literature to provide an overview of the pathogenesis, progression, management, and prevention of this rare complication.

## 2. Subjects and Methods

### 2.1. Participants

This is a retrospective clinical study of 1675 patients who underwent strabismus surgery between 2010 and 2016 at the Department of Ophthalmology, Xiangya Hospital of Central South University, were included in the present study. The study was approved by the Medical Ethics Committee of the Xiangya Hospital of the Central South University.

### 2.2. Inclusion and Exclusion Criteria

Patients who met the following criteria were included in the study: (i) diagnosed with strabismus and underwent strabismus surgery; (ii) had a clinical examination performed once suspicious manifestations of conjunctival cyst were discovered during follow-up; and (iii) had conjunctival cyst located at the surgical site.

Patients who had other ocular conjunctiva-associated tumors or conjunctival hyperplasia were excluded from the study.

### 2.3. Methods

#### 2.3.1. Surgical Modality

Surgery in all patients was performed by a surgeon with more than 20 years of specialization in strabismus and amblyopia treatment. All adults, as well as adolescents who understood the procedure and were cooperative, were given topical anesthesia (qxybuprocaine hydrochloride eye drops, Benoxil®, Santen Pharmaceutical Co. Ltd., Japen) only. No sedation was used. General anesthesia was given to the remaining patients. Park's conjunctival incision was made in parallel on and below the limbus, and the broken end of the muscle was fixated with the double-loop suture technique by an absorbable suture 6-0 (6-0 Coated Coated Vicryl® absorbable, Ethicon, INC, Ethicon, INC), one suture for one muscle, seamed to the designed site on scleral, followed by suturing of the conjunctival incision with absorbable 8-0 (8-0 Coated Vicryl® absorbable, Ethicon, INC). Postoperative local antibiotics (tobramycin/ofloxacin) and steroid (dexamethasone) eye drops were administered to all patients three times daily for 2 weeks.

#### 2.3.2. Follow-Up

All patients were subjected to a follow-up schedule of 6 weeks, 3 months, 6 months' postsurgery, and every 6 months afterwards. In order to remind the patients of timely follow-up, trained nurses would ask the patients about the condition of the incision, vision status, adherence to the medication regimen, and other types of discomfort at one and three months after surgery. A QR code for access to the surgeon's personal web page at Good Doctor website was provided to every patient to facilitate direct communication with the primary surgeon. The patients or the parents were all informed of possible occurrence of conjunctival cyst and its main symptoms. Once the chief complaint from patients was identified as a conjunctival cyst, an appointment was made at the hospital immediately.

## 3. Results

Seven patients (7 eyes; 7out of 1675), between the ages of 3 years and 8 months and 39 years (mean age of 12.71 years (12.71 ± 12.59 years old)), presented with conjunctival cyst at different time points postoperatively. Strabismus surgery affected 13 recti, including 8 medial and 5 lateral recti, and 3 obliques (all inferior obliques). Conjunctival cysts were located at the nasal in 3 cases and temporal side in other 3 cases and at the fornix with inferior oblique surgery in 1 case. The time of discovery of the conjunctival cyst ranged from 10 days to 6 months postoperatively (the detected days, mean time of 42.57 ± 61.11 days). According to the sequence of operation on muscles, there are 2 cases affected at the first or the only operated muscle, 3 cases affected at the second muscle of the first eye, while 2 other cases affected at the second operated eye, as summarized in Table 1.

### 3.1. Case 1

**Figure 1:**
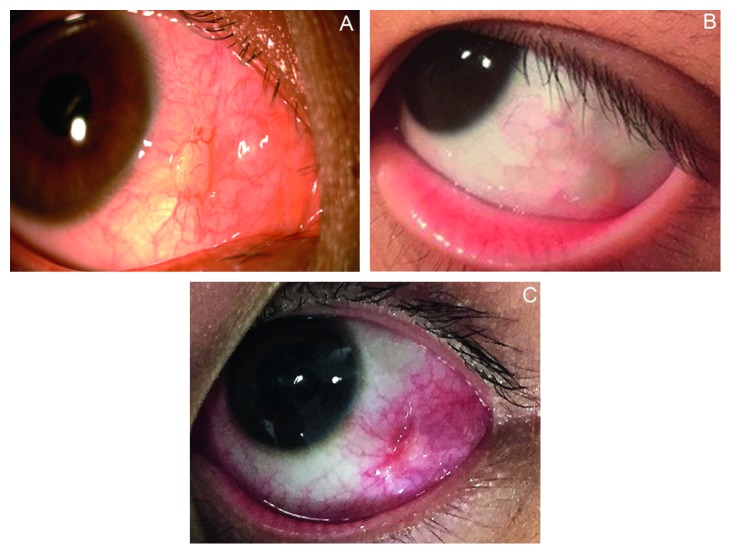
Conjunctival cysts at the left temporal side for Case 1 (a), Case 3 (b), and Case 4 (c).

A 39-year-old female patient underwent surgery under local anesthesia following the diagnosis of concomitant exotropia. In the month following the surgery, the patient complained of a foreign body sensation. A conjunctival cyst was visualized at the middle nasal side of the right eye, with severe conjunctival congestion (Figure 1). Tobramycin and dexamethasone eye drops (Tobradex®, SA Alcon-Couvreur NV, Belgium) were applied 4 times daily for 1 week, together with deproteinized calf blood extract eye gel (Shenyang Xing Qi Ophthalmic Limited by Share Ltd, China) for 2 weeks. The conjunctival cyst resolved in the next 1 month.

### 3.2. Case 2

A 10-year-old male patient underwent strabismus surgery twice (in 2009 and 2015) under general anesthesia following the diagnosis of concomitant exotropia. At 2 weeks postoperatively in 2015, the patient complained of redness affecting the left eye (the second operated eye) and a conjunctival cyst was found at the nasal side. Postoperative medication was further administered for one week, and the cyst resolved in the 1st month follow-up examination.

### 3.3. Case 3

A 4-year-old male patient underwent surgery under general anesthesia following the diagnosis of concomitant exotropia. There weeks postoperatively, his mother found a hyaline cyst of the conjunctiva at the inferior temporal right eye next to the fornix, without evidence of congestion. The patient showed no obvious discomfort, and no specific treatment was applied. At the 5-year follow-up, no change has been reported (Figure 1).

### 3.4. Case 4

A 14-year-old male patient underwent surgery under local anesthesia following the diagnosis of concomitant exotropia. Two weeks later, a conjunctival cyst was found at the left temporal side, with evidence of conjunctival congestion. Tobramycin and dexamethasone eye drops and ointment (Tobradex, SA Alcon-Couvreur NV, Belgium) were applied for 1 week, and the cyst remained unresolved. The patient stopped treatment by himself. Although he showed no symptoms, exploratory surgery of the cyst was performed at the 6th week follow-up (Figure 1). No purulent fluid was detected in the cyst, and mild necrosis was found in the adjacent soft tissues; the suture was not fully absorbed. The suture segment was completely removed and tested negative in bacterial culture.

### 3.5. Case 5

**Figure 2:**
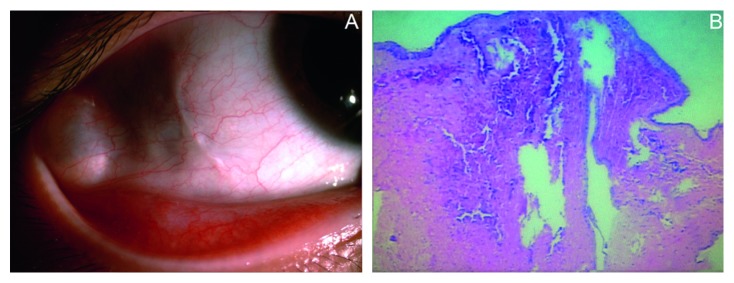
(a) Conjunctival cyst at the right temporal side in Case 5; (b) histopathological picture of right conjunctival cyst.

A 15-year-old female patient underwent surgery under local anesthesia following the diagnosis of concomitant exotropia. Six months postoperatively, the patient complained of foreign body sensation, and a conjunctival cyst, 5 × 5 mm, was found at the right middle temporal side (Figure 2). The cyst was surgically excised 2 years postoperatively. Pathological results showed that the cyst wall was coated with stratified squamous epithelium, with fibrous connective tissue in the cyst cavity (Figure 2).

### 3.6. Case 6

**Figure 3:**
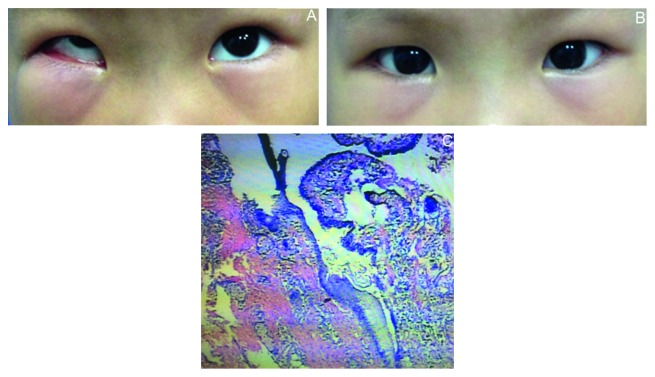
(a) Conjunctival cyst at right inferior fornix in Case 6; (b) four months after surgical excision of conjunctival cyst; (c) histopathological picture of conjunctival cyst.

A 3-year, 8-month-old female patient underwent disinsertion of right inferior oblique and general anesthesia and following the diagnosis of “right superior oblique muscle paralysis.” During the first postoperative month, the patient's mother found her eye showed a restricted up-gaze and limited inferior turn (Figure 3), and swelling of the lower right eyelid was also evident due to the massive size of the cyst. But the patient did not report any discomfort. Conjunctival cyst was found at the inferior fornix conjunctiva. A B-ultrasound scan found cystic degeneration area, irregular in shape, at the subcutaneous region of the lower right eyelid, with clear boundaries, intracystic compartments, and multiple uneven medium to strong echoic masses. Computed tomography (CT) images revealed irregular high-density foci inferior and external to the right eyeball, with uneven internal density and no enhanced signal. Furthermore, signs of evident compression and superior dislocation of the right eyeball and optic nerve were present, as well as an intact eye ring without bone damage. The image diagnosis was recommended as “hematoma considered.” Local application of Levofloxacin eye drops (0.5%, Santen Pharmaceutical Co. Ltd., Japen) combined with tobramycin and dexamethasone eye drops (Tobradex, SA Alcon-Couvreur NV, Belgium) 3 times per day for 2 weeks, resulted in no relief. The cyst was then surgically excised at 4 months postoperatively (Figure 3). Intraoperative findings showed a cystic mass on the surface of the right inferior rectus, with intact cystic wall enclosing caviar-like particles and transparent cystic fluid. The dimensions of the cyst were approximately 6 × 5 × 4 mm, and it was not tightly attached to adjacent tissues. Pathological results revealed a cyst wall coated with stratified squamous epithelium, with fibrous connective tissue in the cyst cavity. The diagnosis of a benign conjunctival cyst was made (Figure 3). Gram staining revealed occasional G^−^ bacilli, but bacterial culture was negative.

### 3.7. Case 7

**Figure 4:**
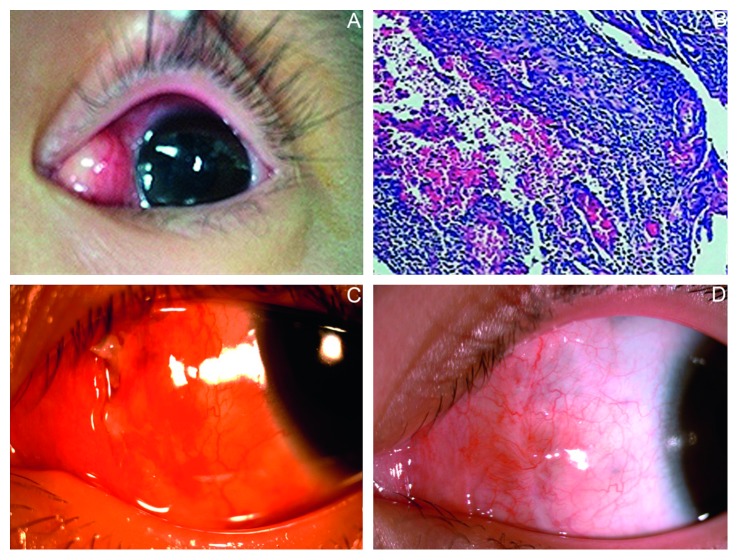
(a) A 7 × 8 mm conjunctival cyst at the left nasal side prior to exploratory surgery in Case 7; (b) postoperative histopathological picture; (c) suture extending out of incision at the left side; (d) wound resolved after left conjunctival suture removal.

A 4-year-old female patient underwent surgery under general anesthesia following the diagnosis of concomitant exotropia. Ten days following the strabismus surgery, the patient showed subconjunctival cyst in the left eye, without evident symptoms (Figure 4). Surgical exploration discovered thin purulent fluid in the subconjunctival cyst, with no evident capsule and an unclear boundary mostly made up of necrotic soft tissue. The broken end of medial rectus was firmly attached to the sclera surface (surgical design: 5 mm posterior insertion) and the initial muscle suture (6-0 Coated Vicryl absorbable, Ethicon, INC) was intact but loose; therefore, it was removed. A portion of necrotic tissue was extracted for bacterial and fungal culture tests, as well as a pathological test which revealed (left subconjunctival) chronic suppurative inflammation (Figure 4). Postoperative bacterial culture was positive for methicillin-resistant Staphylococcus aureus (MRSA) infection. According to a drug sensitivity test, vancomycin (0.25 g, q8h) was administered via intravenous drip, combined with local antibiotic and corticosteroid eye drops (Tobradex, SA Alcon-Couvreur NV, Belgium). Five days postoperatively, scant mucous discharge was present at the conjunctival incision suture (Figure 4). Therefore, the conjunctival suture was removed. At the first postoperative follow-up 1 month after the surgery, a complete recovery of conjunctival incision (Figure 4) with right binocular alignment was shown.

## 4. Discussion

We can find sporadic reports of surgical implantation cysts involve patients who had strabismus surgery [12], retinal detachment surgery with scleral buckling [13], or previous enucleation [14]. Conjunctival cyst is a rare complication of strabismus surgery, with a reported incidence of 0.25% [15]; in our study, we detected 7 cases in 1675 patients, and the rate of detection is 0.4%, which might not be the actual incidence rate of conjunctival cysts. There are several types of conjunctival cyst following surgery: conjunctival epithelium implantation, subconjunctival abscess, chronic granuloma, and conjunctival stress edema [6].

It is generally believed that conjunctival epithelium implantation was the main cause for conjunctival cyst after strabismus surgery. Our observation reported similar results. Three cases (Case 5, Case 6, and Case 7) were confirmed by pathological analysis to be epithelial cysts, which may be associated with conjunctival epithelium implantation. One case (Case 3) had a persistent existence of conjunctival cysts for several years, and the cysts did not resolve after antibiotic eye drops and anti-inflammatory treatments. It is highly possible that conjunctival epithelium implantation played a major role in these cases.

Khan et al. [16] reported a misdiagnosis of infected epithelial inclusion cyst and proposed that presumed subconjunctival abscess after strabismus surgery could all be infected epithelial inclusion cyst. In our study, the pathological section of Case 7 showed the cyst wall was composed of epithelium, and a history of living in kindergarten shortly after operation, which might indicate the possibility of an exogenous infection with epithelial encapsulated cyst formation. Song et al. [12] suggested that a massive conjunctival inclusion cyst may form rapidly when serious infection, such as orbital cellulitis, endophthalmitis takes place due to severe pollution or immune hypofunction. Several groups reported orbital cellulitis occurred days after strabismus surgery [17–19].

Surgery is usually a frequent cause of acquired infection. In the seven cases analyzed in this study, all strabismus surgeries were performed in a laminar flow operation room, and antibiotic eye drops were administered for 3 days before surgery and two weeks after surgery as a precaution. Also, a routine preoperative rinse of the conjunctival cyst was performed with povidone-iodine antibacterial eyewash. Therefore, we suggest a possible cause of early-stage (first month after operation) inflammation is suture response, which is a pathological reaction to suture material in susceptible individuals. A foreign body may induce an immunological rejection, or inflammation, or other factors resulting in a suture response. It has been reported that Vicryl absorptive suture can cause a serious response in the early period after suturing [20]. Absorptive suture is made by multistrand cross knitting and multistrand braiding around one main line, which makes it susceptible to bacterial attachment.

In their randomized, controlled study, Eustis and coworkers [21] found a higher than estimated incidence of needle and suture contamination after strabismus surgery, a 28% bacterial contamination rate for sterile sutures, which is close to the 15–25.2% contamination rate reported by Olitsky et al. [22] and Carothers and coworkers [23]. Even though absorptive sutures used in strabismus surgery are stored in sterile packages, they can touch the eyelashes and skin during operation and get contaminated by germs (mostly conditional pathogenic bacteria) from the hair follicles adjacent to the incision.

A different study found that the same bacterial colony causes infection post cataract surgery as the one residing in the patient's extraocular tissue [24]. Also, the possibility of bacterial contamination of the operative environment cannot be excluded either. During surgery, the 6-0 Vicryl absorbable suture is preset at the broken end of muscle; its distal end can touch the margins of the eyelid and the eyelashes, even the regions outside of the operative field. In agreement with this hypothesis, coagulase-negative *Staphylococci* were found on the eyelid and eyelashes in a previous study [23]. In Cases 4 and 7, nonabsorbent suture segment was visualized floating in the cystic fluid during resection of the conjunctival cyst. The culture of the suture segment was negative for Case 4 and positive for MRSA for Case 7. The negative bacteria culture result of Case 4 could be affected by the effective antibiotic eye drops used before surgery.

Common pathogenic bacteria causing infection after strabismus surgery include *Staphylococcus aureus*, *Staphylococcus epidermidis*, *Streptococcus pneumoniae*, and *Haemophilus influenzae*. Statistical data from a comprehensive hospital trial (all departments) revealed that the primary pathogens causing acquired hospital infection include *Escherichia coli* [24], *Staphylococcus aureus*, and *Proteus mirabilis* [23]. Among them, anaerobic bacteria, such as *Pseudomonas aeruginosa* and MRSA, are particularly common in a surgery setting. In a large-scale survey, Kivlin and Wilson [17] reported 308 forms of postoperative infection after strabismus surgery, which was carried out at multiple hospitals affiliated with the American Academy of Strabismus and Pediatric Ophthalmology. *Staphylococcus aureus* was isolated in 56% of 25 cases. Among the 25 cases, sub-Tenon's abscess was found in 3 cases. All three cases experienced symptoms in the first week after the surgery, with positive cultures for *Proteus mirabilis* and *Staphylococcus aureus* obtained. Since 1988, MRSA incidence has been on the rise. One study uncovered type II coagulase as the only coagulase type in MRSA, which is also one of the most common colonies causing acquired hospital infection [25]. Since the late 1990s, the incidence rate of community-associated MRSA (CA-MRSA) has increased on an annual basis, particularly in children and adolescents [18, 19], mainly because of frequent physical contact in schools and kindergartens. In order to prevent postoperative infection, Ing and coworkers [26] highlighted the importance of personal hygiene in patients undergoing eye surgery. Case 7 is a child who developed conjunctival cyst two days after returning to kindergarten, with a positive MRSA culture of the excised specimen. In this case, the suture was found separated free from the muscle with some necrotic adjacent tissue around. Therefore, we inferred that reaction between the suture and the muscle might be the primary cause of inflammatory. During the process, inflammatory secretion accumulated and the cyst got enlarged, subsequently followed by a thinning or cracks of the wall, which made it susceptible for exogenous MRSA infection.

Another risk factor is the duration of surgery. A longer procedure entails longer exposure of the instruments and suture to the air and thus is accompanied with a greater chance of bacterial infection at the incision site [23]. In our study, 7 diseased eyes from 7 cases were categorized according to the sequence of muscle which was operated on; the first muscle was affected in 2 cases (the first and sole operated muscle in 1 case), while the second or above muscle was affected in 5 cases. We inferred that infection might be more common in the muscle of the eye which is operated later. However, the small sample size and use of routine antibiotic eye drops before surgery highlighted the necessity of future study to confirm our hypothesis.

Al-Shehah [27] noted that conjunctival cyst tends to enlarge gradually and more treatment is required to eliminate the enlargement. Therefore, he recommended early excision. Hawkins recommended thermal cautery under the ophthalmic slit lamp [28], while some specialists tried ethanol injection into the cyst [10]. Other studies documented the role of povidone-iodine in the reduction of suture colonization [29] while antibiotic-embedding of the suture was reported to reduce postoperative infection after strabismus surgery [21].

The limitations of our study include a retrospective data set obtained only from one hospital unit and one surgeon. Furthermore, not all patients were adhering to the follow-up schedule. It is possible that some conjunctival cysts were not discovered due to their tiny size, hidden position, or spontaneous regression in the early short period of time after surgery. Therefore, the actual incidence rate of conjunctival cysts after strabismus surgery is likely to be higher than what is reported here. Even though most of them are not considered serious complications, conjunctival cysts can interfere with postoperative recovery and need multiple intervention in severe cases, such as Case 6 and Case 7.

In summary, conjunctival cyst is a rare complication of strabismus surgery. Conjunctival epithelium implantation is the primary cause, and infection might exaggerate the situation. Suture contamination, poor personal hygiene, or longer duration of surgical procedure increases the possibility of infection.

## Figures and Tables

**Table 1:** Patient characteristics.

Case number	Sex	Age (yrs)	Surgical modality	Cyst site	Discovery time	Primary symptoms	The operated sequence of affected muscle	Treatment and prognosis	Pathology	Bacteria culture
1	Female	39	Right lateral rectus recession and medial rectus resection	Middle right nasal	1 month	Foreign object sense	Sole surgical eye, the 2nd muscle	Spontaneous remission	NA	NA

2	Male	10	Right lateral rectus recession and medial rectus resection in 2009, bilateral inferior oblique myectomy + medial rectus recession in 2015	Left subnosal	2 weeks	Redness	The 2nd surgical eye	Spontaneous remission	NA	NA

3	Male	4	Right lateral rectus recession and medial rectus resection	Inferior temporal fornix	>20 days	Conjunctival cyst found	Sole operated eye, the 1st muscle	Asymptomatic persistent	NA	Negative

4	Male	14	Right medial rectus recession and lateral rectus resection	Right temporal	2 weeks	Foreign body sense and cyst found	The 2nd muscle of operated eye	Excision at 6 weeks postoperative	NA	Negative

5	Female	15	Bilateral rectus recession and right medial rectus resection	5 × 5 mm, middle right temporal	6 months	Foreign body sense and cyst found	The 2nd operated eye	Excision at 2 years postoperative	Squamous epithelium	Negative

6	Female	3	Inferior oblique myectomy	Right inferior fornix	1 month	Restriction of eye movement, swelling of the eyelid.	Sole operated eye, the only muscle	Excision at 4 months postoperative	Dermoid cyst	Negative

7	Female	4	Bilateral medial rectus recession	Left nasal	10 days	Cyst found	The 2nd operated eye, the 2nd muscle	Excision at 6 months postoperative	Inflammation, single-layer epithelium	MRSA+
